# HeartMate 3 for Heart Failure with Preserved Ejection Fraction: In Vitro Hemodynamic Evaluation and Anatomical Fitting

**DOI:** 10.1007/s10439-024-03585-y

**Published:** 2024-07-16

**Authors:** Nina Langer, Andrew F. Stephens, Michael Šeman, David McGiffin, David M. Kaye, Shaun D. Gregory

**Affiliations:** 1 Cardio-Respiratory Engineering and Technology Laboratory (CREATElab), Department of Mechanical and Aerospace Engineering, Monash University, Melbourne, VIC Australia; 2 Victorian Heart Institute, Victorian Heart Hospital, Melbourne, VIC Australia; 3 School of Public Health and Preventative Medicine, Monash University, Melbourne, VIC Australia; 4 The Department of Cardiology, The Alfred Hospital, Melbourne, VIC Australia; 5 Department of Cardiothoracic Surgery, The Alfred, Melbourne, VIC Australia; 6 Victorian Heart Hospital, Melbourne, VIC Australia

**Keywords:** HFpEF, Mechanical circulatory support, Cardiovascular simulator, Acute heart failure, Left ventricular assist device, Anatomical fitting

## Abstract

Heart failure with preserved ejection fraction (HFpEF) constitutes approximately 50% of heart failure (HF) cases, and encompasses different phenotypes. Among these, most patients with HFpEF exhibit structural heart changes, often with smaller left ventricular cavities, which pose challenges for utilizing ventricular assist devices (VADs). A left atrial to aortic (LA-Ao) VAD configuration could address these challenges, potentially enhancing patient quality of life by lowering elevated mean left atrial pressure (MLAP). This study assessed the anatomical compatibility and left atrial unloading capacity using a simulated VAD-supported HFpEF patient. A HeartMate3-supported HFpEF patient in an LA-Ao configuration was simulated using a cardiovascular simulator. Hemodynamic parameters were recorded during rest and exercise at seven pump flow rates. Computed tomography scans of 14 HFpEF (NYHA II–III) and six heart failure with reduced ejection fraction patients were analysed for anatomical comparisons. HFpEF models were independently assessed for virtual anatomical fit with the HM3 in the LA-Ao configuration. Baseline MLAP was reduced from 15 to 11 mmHg with the addition of 1 L/min HM3 support in the rest condition. In an exercise simulation, 6 L/min of HM3 support was required to reduce the MLAP from 29 to 16 mmHg. The HM3 successfully accommodated six HFpEF patients without causing interference with other cardiac structures, whereas it caused impingement ranging from 4 to 14 mm in the remaining patients. This study demonstrated that the HM3 in an LA-Ao configuration may be suitable for unloading the left atrium and relieving pulmonary congestion in some HFpEF patients where size-related limitations can be addressed through pre-surgical anatomical fit analysis.

## Introduction

Heart failure (HF) is a rising global disease, having a prevalence of more than 64 million individuals worldwide [1]. The type of HF is split almost evenly: approximately 50% with reduced ejection fraction (HFrEF) and approximately 50% with heart failure with preserved ejection fraction (HFpEF) [2]. The diagnosis of HFpEF is often delayed, and the treatment options are limited [3]. HFpEF patients can be stratified based on various criteria, for example by clinical representation, hemodynamic changes, epidemiology or hospitalization criteria [4]. Based on the etiology of HFpEF, the structural changes associated with the disease are different [5]. For example, some HFpEF patients show a variety of abnormal geometries of the left ventricle (LV) [6, 7] and are likely to have a smaller LV cavity compared to healthy individuals and HFrEF patients [8]. The left atrial pressure (LAP) in the majority of these patients is typically elevated [9] and the patients are associated with long-term mortality [10].”

Patients with HFrEF may be treated with a durable ventricular assist device (VAD) for mechanical circulatory support, typically as a bridge to transplant or destination therapy. However, VADs are typically not implanted in patients with HFpEF and a smaller LV cavity [11–16], as the changes in cardiac geometry increase the risk of occlusion of the VAD inflow cannula and, consequently, reduced capacity for mechanical support [11]. An alternative inflow cannulation site for a VAD is the left atrium (LA), which has been demonstrated to decrease LA pressure [17, 18]. This approach may be more suitable for these HFpEF patients, given their abnormal cardiac geometry and need to unload the LA.

LA inflow cannulation with the only clinically available durable VAD, the HeartMate 3 (HM3—Abbott Laboratories, Abbott Park, Illinois), has been used to support patients with HFrEF in the past, yet there are no reports of how this VAD might fit with the anatomical structures of a HFpEF patient [19]. The feasibility of HM3 implantation in the LA of HFpEF patients thus requires further investigation and can be aided by the established technique of virtual anatomical fitting using patient-specific 3D models generated from computed tomography (CT) images [20–22].

This study aimed to assess the suitability of the HM3 for mechanical circulatory support of HFpEF patients, focusing on in vitro evaluation of the hemodynamic performance and virtual anatomical fit. Hemodynamic performance was assessed via the capacity of the HM3 to unload the left heart during rest and exercise with alterations in pump speed, while anatomical fit was assessed via the ability to fit the HM3 device within the chest and the inflow cannula within the LA, without impingement on other anatomical structures.

## Materials and Methods

### Hemodynamic Evaluation

A previously developed mechanical cardiovascular simulator [23], including the heart, systemic circulation, and pulmonary circulation, was used to evaluate the hemodynamic performance of the HM3 in a simulated HFpEF patient. The heart chambers were modelled by clear vertical polyvinyl chloride cylinders (ALSCO, Atlanta, GA, USA), connected by tee junctions. The aortic, mitral, pulmonary and tricuspid valves were modelled by mechanical umbrella valves. A series of regulators (ITV1030-31N2BL5-X88, SMC Corporation, Tokyo, Japan) and solenoid valves (VT325-035DLS, SMC Corporation, Tokyo, Japan) controlled ventricular systole and diastole by switching between delivering compressed air during systole and controlled venting to atmosphere during diastole to allow passive filling of the ventricles. The Starling response of the LV was controlled manually by adjusting the LV contraction based on the left ventricular end-systolic pressure and volume (LVESP, LVESV). In the right ventricle, a Starling response, described by Gregory et al. [20, 23] automatically adapted the right ventricular contraction based on the preload measured in the ventricular chamber.

**Fig. 1:**
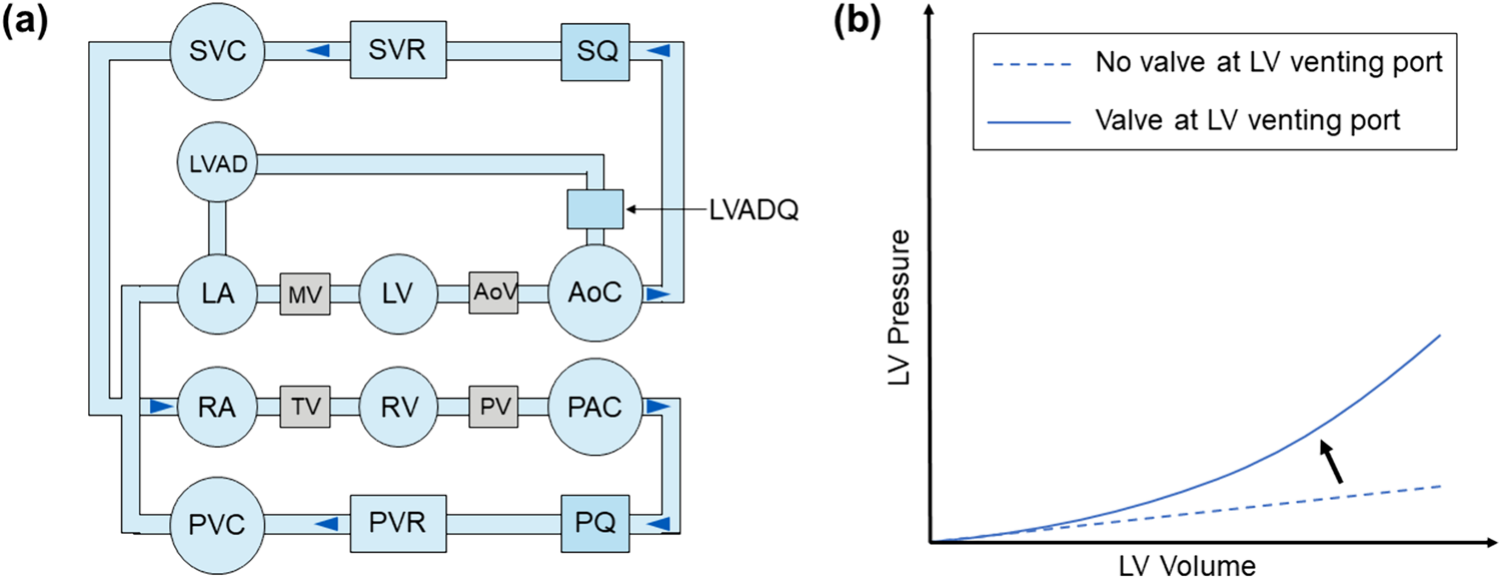
**a** Schematic of the cardiovascular simulator with a left ventricular assist device in left atrial to aortic configuration: *SVC, PVC* systemic and pulmonary vascular compliance, *SVR, PVR* systemic and pulmonary vascular resistance, *SQ, PQ* systemic and pulmonary flow sensors,* LA, RA* left and right atria, *MV, TV* mitral and tricuspid valves, *LV, RV* left and right ventricles, *AoV, PV* aortic and pulmonary valves, *AoC, PAC* aortic and pulmonary compliance, *LVAD* left ventricular assist device, *LVADQ* left ventricular assist device flow sensor, **b** illustration of the left ventricular end-systolic and diastolic pressure–volume relationship modification by amending the Starling mechanism and the implementation of a valve between the left ventricle and atmosphere: *LVPRV* left ventricular pressure–volume relationship

The previous setup, described by Gregory et al. [23], produces a linear and shallow LV end diastolic pressure volume relationship (EDPVR) due to the constant diameter of the vertical LV chamber and the venting port of the LV solenoid valve. To simulate the LV EDPVR of a HFpEF patient, a direct-acting 2-way solenoid control valve (Type 2836, Buerkert, Ingelfingen, Germany) was placed at the venting port of the LV controlling discharge to atmosphere. The valve was controlled with a PWM signal between 0 and 10 V at 180 Hz. By changing the voltage applied to the valve, the passive filling of the LV was controlled, which subsequently altered the shape of the LV EDPVR and enabled a steeper slope at higher pressures. Figure 1a illustrates a schematic of the described test rig.

The data was acquired with a dSPACE 1202 MicroLabBox (dSPACE GmbH, Paderborn, Germany). TruWave disposable pressure sensors (Edwards Lifesciences, Irvine, CA) were used for pressure measurement, and clamp-on ultrasonic sensors were used for flow measurement (em-tec BioProTT 3/8″ × 1/8″ for LVAD flow; 3/8″ × 1/8″ for systemic flow; 1″ × 1/8″ for pulmonary flow; SonoTT DigiFlow board, em-tec GmbH; Finning; Germany). The flow measurements were averaged over five seconds. Linear magnetic level sensors (MTL4-650MM, Miran Technology Co., Shenzhen, China) were utilized for measuring left and right ventricular volumes. A water/glycerol mixture (60/40% w/w) was used for the circuit fluid, having a viscosity of 3.5 mPa s and a density of 1100 kg∙m^−3^ at 22 °C, similar to blood at 37 °C.

The cardiovascular simulator was tuned to mimic the hemodynamic conditions of an end-stage HFpEF patient (NYHA II–III) in rest and exercise, as presented by Kaye et al. [21] and Wessler et al. [22]. To achieve these hemodynamic profiles, the Starling response and the LV venting valve control were amended to follow a steepened LV end-systolic pressure–volume relationship (ESPVR) and EDPVR curve, described by Kaye et al. [21] (Fig. 1b). Pressure waveforms of a simulated HFpEF patient in rest and exercise were recorded. For validation of the implemented LV EDPVR, pressure–volume loops at three different LV volumes were recorded, a curve fitted to the LVEDP and compared to the patient data presented by Kaye et al. [21] at rest and exercise, respectively.

To model an exercise condition, the heart rate was increased from 70 to 90 bpm, and systemic and pulmonary vascular resistance (SVR, PVR) were amended from 1600 and 170 to 1250 and 130 dynes seconds cm^−5^, respectively. Furthermore, a left and right-sided atrial kick was implemented, and an additional 400 mL of fluid was shifted to the heart and arterial system from the venous reservoir [24].

The HM3 was positioned between the LA and the aorta (Ao), connected with 390 mm of ½″ tubing. Pressure and flow rate parameters in rest and exercise conditions were recorded at seven different HM3 support levels: no support (pump clamped and turned off), 1, 2, 3, 4, 5 and 6 L/min support through variations in HM3 speed with washout turned on. Hemodynamic changes were evaluated in the context of relieving HF symptoms in HFpEF patients. A waiting period of approximately one minute was observed at each state before commencing data recording, allowing the test rig to stabilize. Five cardiac cycles were recorded at each state. Measures for evaluating pump support included aortic pressure (AOP), left ventricular pressure (LVP), left atrial pressure (LAP), pulmonary artery pressure (PAP), right ventricular pressure (RVP), right atrial pressure (RAP), cardiac output (CO), and pump speed. The raw data was processed with an 8 Hz filter.

### Virtual Fitting

3D-virtual fitting was completed using contrast-enhanced cardiac CT scans of 14 patients with HFpEF (NYHA II–III). The scans were obtained during routine clinical examination and de-identified for this study. The median age of the HFpEF patients was 64 ± 9 years. Of the 14 patients, eight were male, and six were female.

**Fig. 2:**
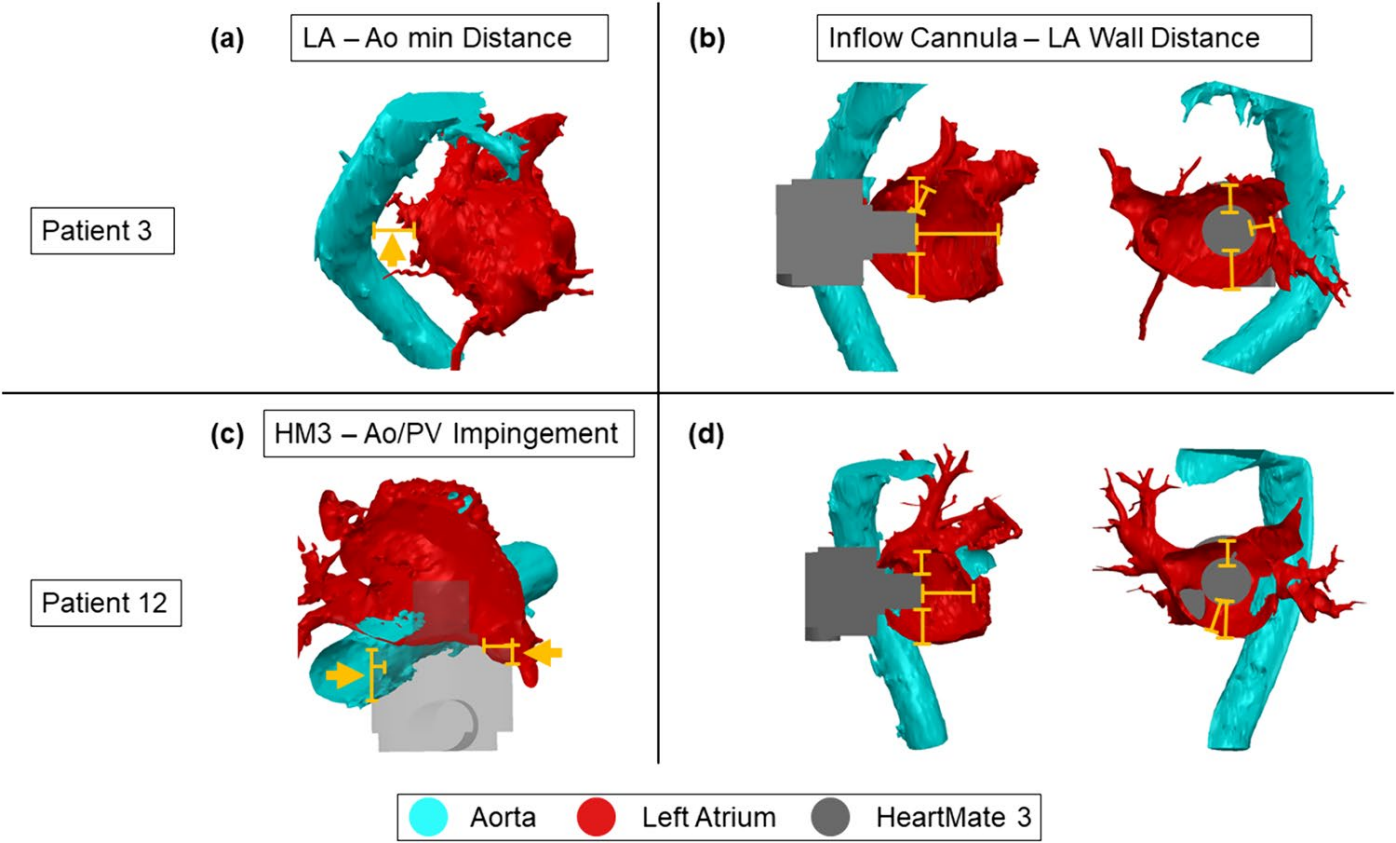
Virtual fitting of the HeartMate 3 in the left atrium of two heart failure with preserved ejection fraction patients. **a** Measurement of left atrium—aorta minimal distance, no impingement of other structures (patient 3); **c** measurement of impingement of the HeartMate3—aorta/pulmonary veins (patient 12); **b, d** Measurement of minimal and maximal distance between the HeartMate3 inflow cannula and the left atrial walls (patient 3 and 12)

The scans were imported into Mimics (Materialise GmbH, Leuven, Belgium). The greyscale of the images was manually adjusted, and masks were created with the custom grey value threshold tool (adjusted according to each patient). The resulting mask contained all regions where blood with contrast fluid could be identified and was subsequently divided into different regions of the heart using the split mask tool. Based on the grey value of the CT images, manual mask refinements were applied on the mask to consider an uneven distribution of contrast fluid in the blood-filled areas, focusing on the geometry of the LA and the Ao. The LA volume and the smallest distance between the LA and descending Ao, which provides information about geometrical restrictions for pump implantation, were measured in 3-matic (Materialise GmbH, Leuven, Belgium). The pulmonary veins were cut off the 3D LA models to measure the LA volume but remained for virtual pump fitting. The measured volumes and distances and the respective median within the patient cohorts were noted and compared against each other. Figure 2 illustrates how the measurements were taken.

A 3D model of the HM3, with a cylinder modelling the sewing ring, was overlayed on the HFpEF heart models and placed with the inflow fully implanted and the sewing ring butted up against the LA. The position of the pump was adjusted for each patient to ensure the pump outflow was directed towards the descending Ao and that the pump had minimal interference with the Ao, pulmonary veins, pulmonary arteries and the right atrium. Based on this model, the virtual fitting of the HM3 was assessed quantitatively. A good anatomical fit was considered as having a pump placement without any interference with rigid body parts or other heart structures. The minimum and maximum distance between the inflow cannula tip and the LA wall, as well as the maximum impingement from the HM3 in other heart structures such as the descending Ao, pulmonary veins, or right atrium, was measured in SOLIDWORKS (SolidWorks Corp., Dassault Systèmes, Vélizy-Villacoublay, France).

## Results

### Hemodynamic Evaluation

**Fig. 3:**
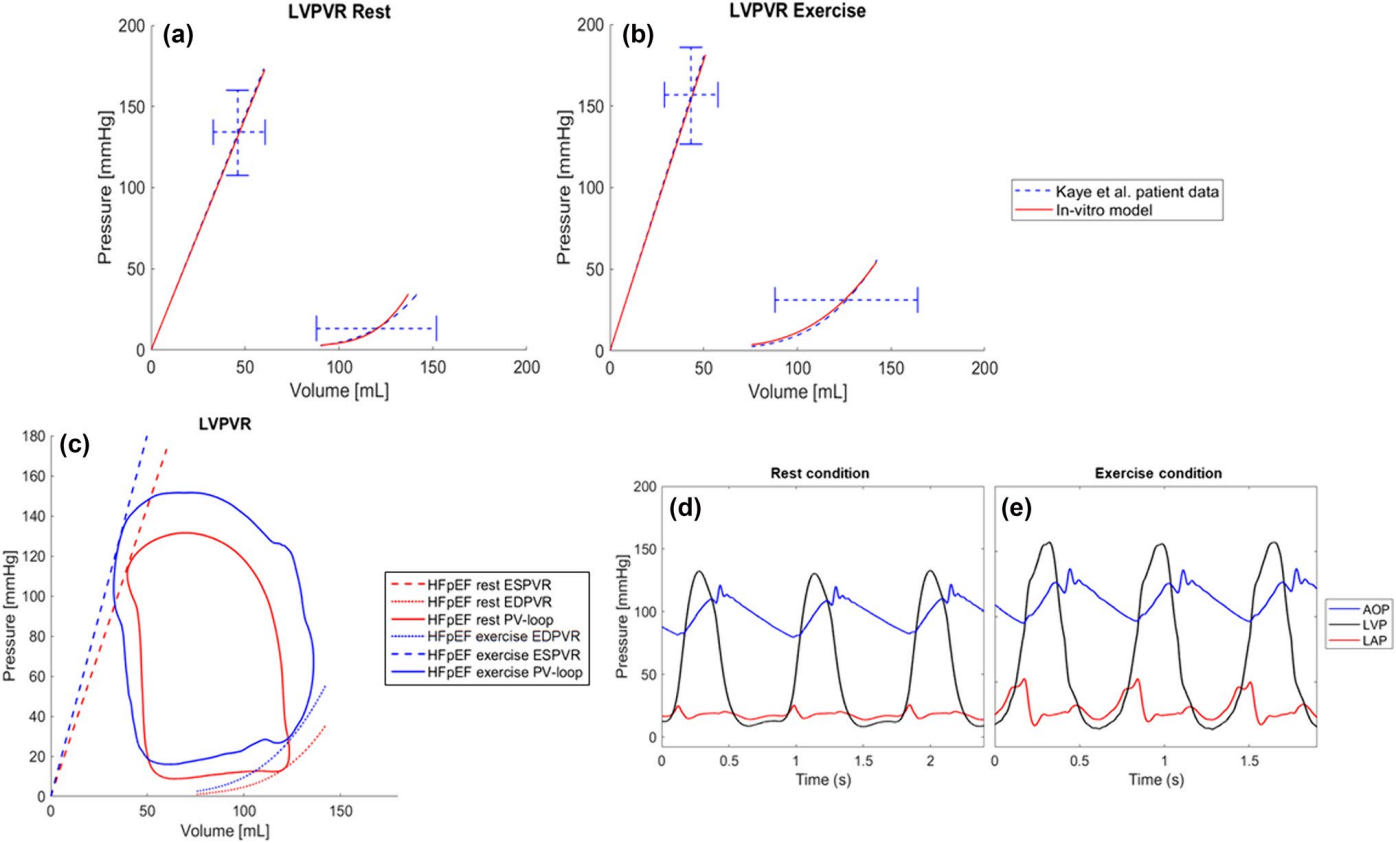
Left ventricular pressure–volume relationship of the simulated heart failure with preserved ejection fraction patient in the in-vitro model (red) compared to patient results presented by Kaye et al. [21] (blue) at rest (**a**) and exercise (**b**), left ventricular pressure volume loops (**c**) and systemic pressure waveforms in rest (**d**) and exercise (**e**) condition of the simulated HFpEF patient without HeartMate 3 support: *HFpEF* heart failure with preserved ejection fraction, *LVPVR* Left ventricular pressure volume relationship, *PV-loop* pressure–volume loop, *EDPVR* end diastolic pressure volume relationship, *ESPVR* end systolic pressure volume relationship, *AOP* aortic pressure; *LVP* left ventricular pressure, *LAP* left atrial pressure

The addition of the LV venting valve created a HFpEF simulation with a steeper EDPVR and ESPVR curve. Figure 3a, b illustrate the target LV ESPVR and LV EDPVR reported by Kaye et al. [21] in blue and the recorded data from the in-vitro model in red. This change subsequently increased mean LAP (MLAP) at rest and exercise from 9 and 17 (healthy) to 15 and 29 mmHg (HFpEF), respectively (Fig. 3d, e). Hemodynamic parameters from HFpEF patients reported in the literature and of the modelled HFpEF patients are shown in Table 1. The reported parameters, except from MRAP, are within the standard deviation of the literature values.

**Table 1:** Hemodynamic parameters of heart failure patients with preserved ejection fraction from the literature [21] and from the in-vitro model at rest and exercise: *HR* heart rate, *MLAP* mean left atrial pressure, *MAP* mean arterial pressure, *SVR* systemic vascular resistance, *MRAP* mean right atrial pressure, *MPAP* mean pulmonary artery pressure; *PVR* pulmonary vascular resistance

Parameter	HFpEF literature		HFpEF in-vitro model	
	Rest	Exercise	Rest	Exercise
HR [bpm]	65 ± 13	101 ± 24	70	90
MLAP [mmHg]	13 ± 4	31 ± 5	15	29
MAP [mmHg]	101 ± 18	117 ± 22	97	114
SVR [dynes∙seconds∙cm^−5^]	1664 ± 528	1256 ± 480	1600	1250
MRAP [mmHg]	8 ± 3	16 ± 4	9	10
MPAP [mmHg]	23 ± 6	43 ± 7	24	39
PVR [dynes∙seconds∙cm^−5^]	168 ± 80	128 ± 64	168	130

The LV ESPVR and LV EDPVR in the HFpEF model exhibited a steeper slope compared to those of a healthy patient. Specifically, the LV ESPVR curve in the HFpEF model demonstrated a slope of 2.85 mmHg/mL at rest and 3.55 mmHg/mL during exercise.

**Fig. 4:**
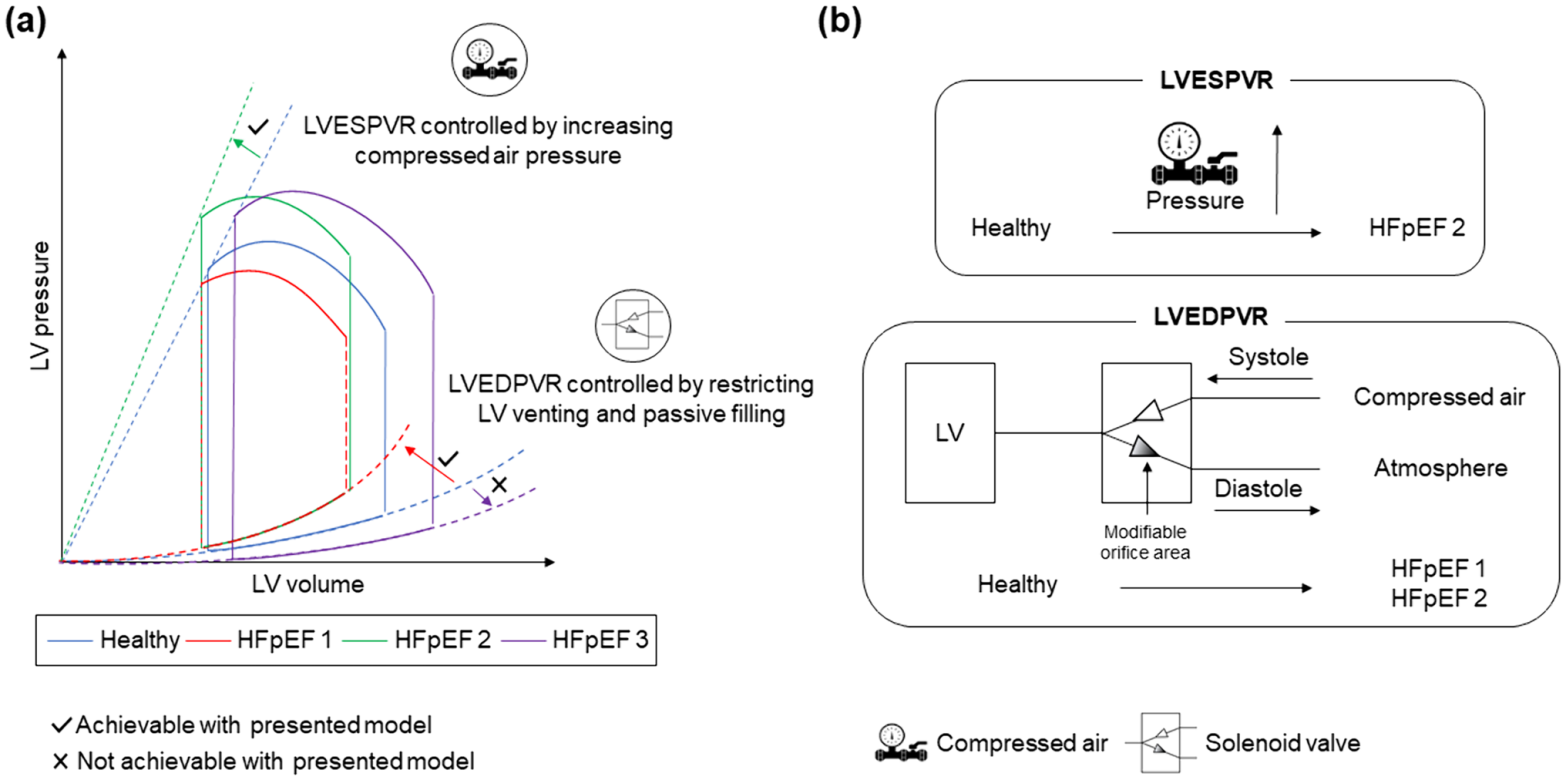
Major heart failure with preserved ejection fraction phenotype related changes in left ventricular pressure volume relationships (**a**), and the set-up changes to simulate the different phenotypes (**b**); *HFpEF* heart failure with preserved ejection fraction, *LV* left ventricle, *LVESPVR* left ventricular end systolic pressure volume relationship, *LVEDPVR* left ventricular end diastolic pressure volume relationship, *atm* atmosphere

The cardiovascular simulator is able to simulate upwardly shifted LVEDPVR by restricting the LV venting and passive filling. Independently, the cardiovascular can simulate increases and decreases in LVESPVR by controlling the compressed air pressure utilized to simulate ventricular contraction. It cannot simulate downward shifted LVEDPVR (Fig. 4).

**Fig. 5:**
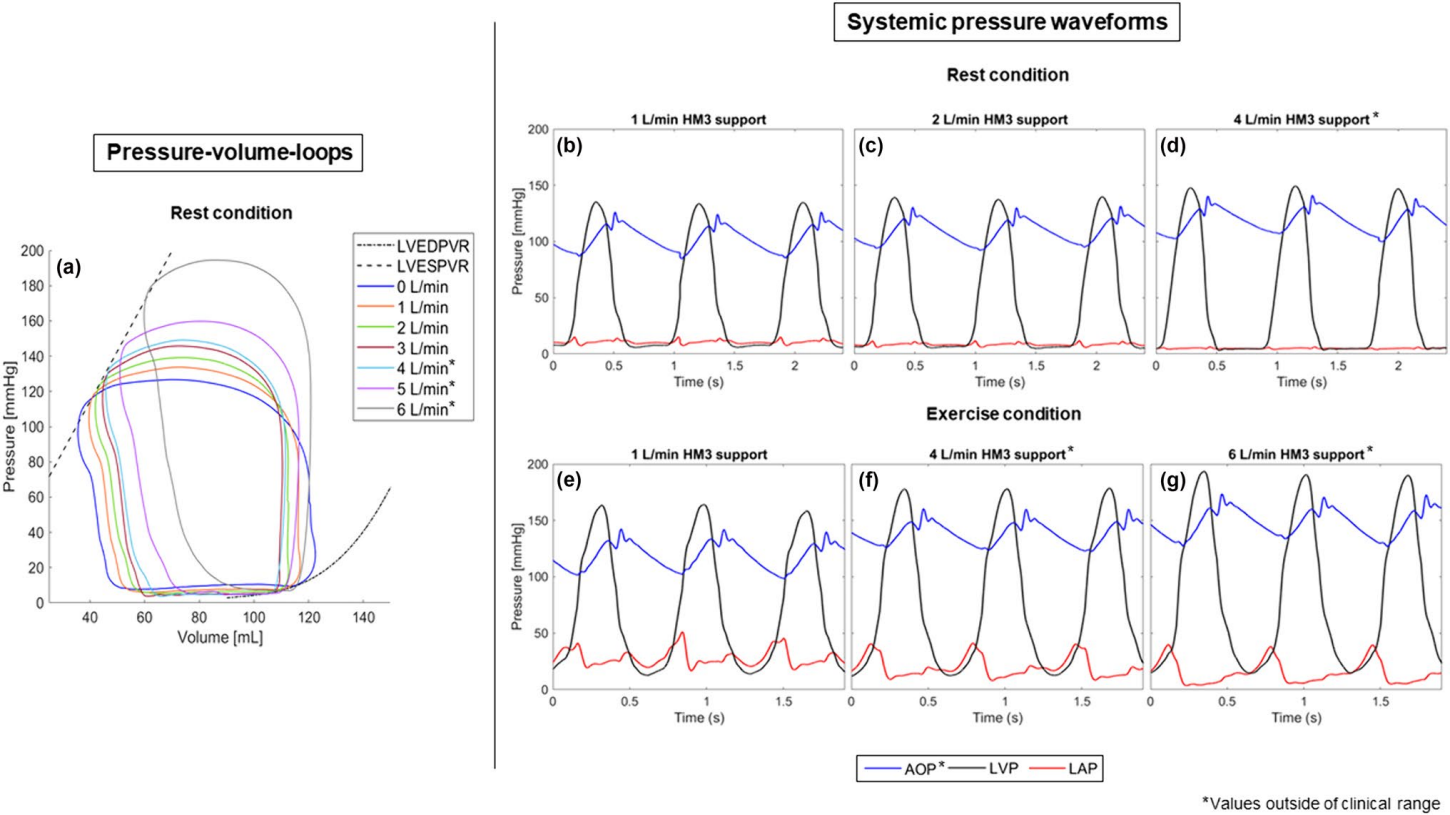
Left ventricular pressure–volume relationship in the simulated patient with heart failure with preserved ejection fraction with pump support at 0, 1, 2, 3, 4, 5, and 6 L/min at rest (**a**) and systemic pressure in a patient in rest (**b–d**) and exercise (**e–g**) condition with heart failure with preserved ejection fraction with 1 L/min (**b, e**), 2 L/min (**c**), 4 L/min (**d, f**), and 6 L/min (**g**) HeartMate 3 support; *LVEDPVR* left ventricular end-diastolic pressure–volume relationship, *LVESPVR* left ventricular end-systolic pressure–volume relationship, *LVP* left ventricular pressure, *LAP* left atrial pressure, *AOP* aortic pressure

At rest, MLAP and mean PAP (MPAP) decreased with higher pump flow rates from 14.8 to 4.9 mmHg (MLAP) and from 24.1 to 16 mmHg (MPAP) at 4 L/min support (Fig. 5). Negative MLAP (− 5.1 mmHg) indicating suction was observed at a pump flow rate of 6 L/min. In contrast, AOP, LVP, mean arterial pressure (MAP) and mean RAP increased with increasing pump flow rates. Intermittent backflow in the pump was observed at pump speeds below 4700 rpm. Increasing pump support led to increased LVESP and LVESV, while a noticeable reduction in left ventricular end-diastolic pressure (LVEDP) and stroke volume was observed as pump support levels increased (Fig. 5).

In exercise, a pump flow rate of 4 L/min reduced MLAP from 28.7 to 19.3 mmHg and MPAP from 39.1 to 31.4 mmHg. Increasing pump flow to 6 L/min to provide improved left atrial unloading resulted in a reduction of MLAP from 28.7 to 16 mmHg and of MPAP from 39.1 to 26 mmHg (Fig. 5). Similar to the rest condition, AOP, LVP, MAP, and mean RAP increased with increasing pump flow and intermittent backflow was recorded at 4800 rpm. The LVESP increased with increasing pump speed, but the LVESV did not increase notably. The LVEDP did not decrease consistently with increasing pump support, and no pronounced variations in stroke volume were evident with increasing pump support. The recorded hemodynamic parameters of the simulated conditions are summarised in Table 2.

**Table 2:** Recorded hemodynamic parameters of a simulated heart failure with preserved ejection fraction patient at rest and exercise at various pump support levels

Pump support [L/min]	Rest							Exercise						
	0	1	2	3	4	5	6	0	1	2	3	4	5	6
CO [L/min]	4.3	4.7	4.9	5.3	5.5	6	7.1	6.6	7.0	7.4	7.7	7.9	8.4	8.8
MLAP [mmHg]	14.8	10.6	8.4	6.2	4.9	1.9	− 5.1	29.2	27.5	24.6	22.0	19.3	17.7	16.0
MAP [mmHg]	96.6*	103.7*	109.6*	113.2*	119.9*	126.9*	159.8*	113.7*	119.1*	123.3*	129.7*	140.9*	143.2*	147.9*
LVSW [W]	1.1	1.1	1	1.1	1	1.2	1.4	1.7	1.7	1.7	1.6	1.7	1.9	1.9
MRAP [mmHg]	9.1	9.2	9.5	9.8	10.1	10.3	10.9	9.9	10.1	10.5	11.0	11.3	11.9	12.1
MPAP [mmHg]	24.1	20.1	18.7	16.7	16.4	13.9	10.0	39.5	38.6	35.6	33.1	31.4	29.7	26.2
Pump speed [rpm]	–	5300	5750	6300	6850*	7550*	8700*	–	5300	5800	6350	6900*	7550*	8350*

Values with * are outside of the clinical range

*HR *heart rate, *CO* cardiac output, *MLAP* mean left atrial pressure, *MAP* mean arterial pressure, *MRAP* mean right atrial pressure, *MPAP* mean pulmonary artery pressure

At rest and exercise, the impact of the HM3 support on the LV stroke work is negligible at low flow rates until 4 L/min. At and above a HM3 support of 5 L/min, the LV stroke work increases.

### Virtual Fitting

The smallest LA volume within the analysed HFpEF patients was 118 mL, while the largest LA volume was 318 mL. The minimal distance between the LA and the descending Ao ranged from 0 to 17.4 mm. All measurements are summarized in Table 3.

**Fig. 6:**
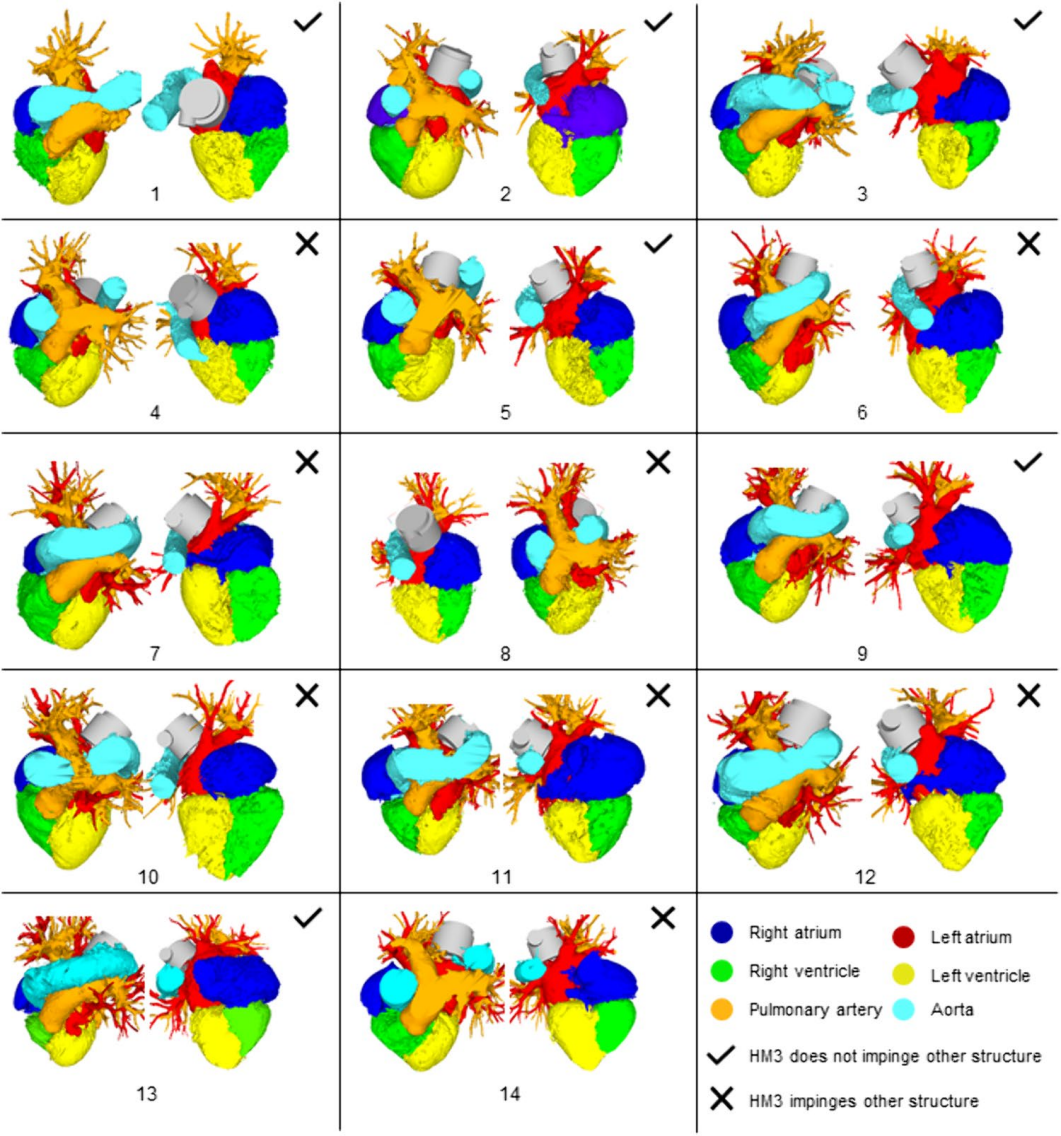
The 3D models of hearts of 14 patients with heart failure with preserved ejection fraction with a HeartMate 3 fitted in left atrial to aortic configuration

**Fig. 7:**
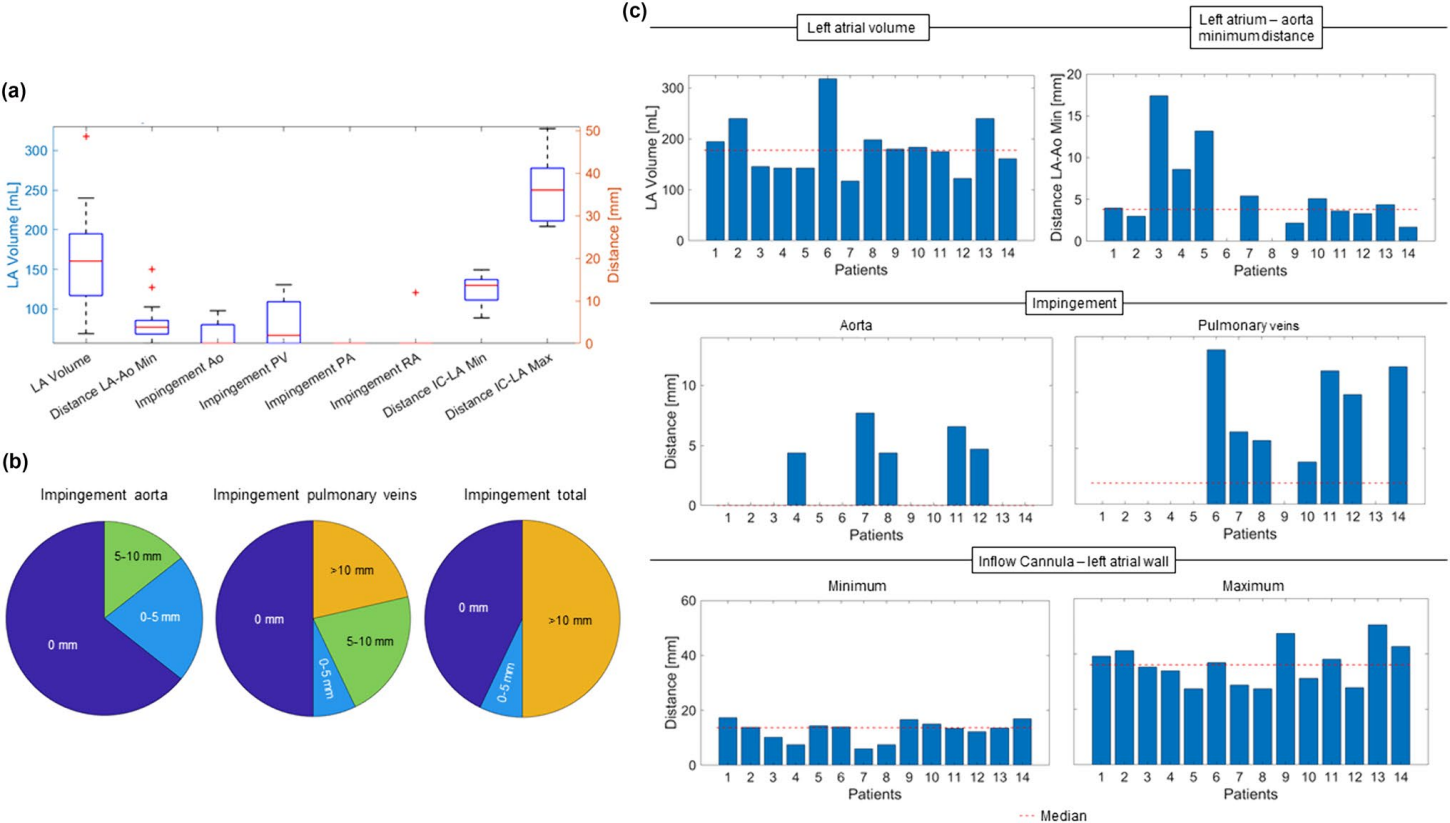
Virtual anatomical fitting results illustrated as boxplots (**a**), impingement of the HeartMate3 of aorta, pulmonary veins and total impingement of aorta, pulmonary veins, pulmonary artery and right atrium in pie charts (**b**), and left atrial volume, minimum distance between the left atrium and the aorta, impingement of the HeartMate3 of the aorta, pulmonary veins and total impingement of aorta, pulmonary veins, pulmonary artery and right atrium, minimum and maximum distance between the inflow cannula and the left atrial wall and the respective median of each parameter as dashed line (**c**): *LA* left atrium, *Ao* Aorta, *PV* pulmonary veins, *PA* pulmonary artery, *rA* right atrium, *IC* inflow cannula

In six of the 14 assessed patients, the HM3 could be positioned without impinging other structures of the heart. In one model, the impingement of the pulmonary veins was 3.8 mm (#10 Fig. 6), and in three patients, the impingement from the HM3 in the pulmonary veins or aorta was between 5 and 10 mm (#7, 8, 12 Fig. 6). In each HFpEF model, the HM3 was fitted without interfering with the ribs. The minimal distance between the inflow cannula and the closest LA wall was 6 mm, with a median of 13.64 mm across all assessed HFpEF patients, based on full insertion (Fig. 6). Figure 6 shows the positioning of the HM3 in the LA in all assessed HFpEF patients and Fig. 7 illustrates the results of the virtual anatomical fitting. More details can be found in the appendix.

## Discussion

Unloading the LA to the Ao with a VAD may be beneficial to relieve HF symptoms in HFpEF patients [18, 25]. In our study, the HM3 was implemented in an in vitro cardiovascular simulation of a HFpEF patient to investigate the hemodynamic impact of VAD support and in a series of virtual patients to assess anatomic fit.

The HM3 in an LA to Ao configuration was found to reduce LAP and LVEDP while increasing the afterload at rest, which is consistent with results reported from in silico studies [17, 25, 26]. The HM3 provided a MLAP reduction at rest of 67% with pump support of 4 L/min, and the MLAP first dropped below 13.2 mmHg at a pump flow rate of 1 L/min, which may be sufficient to decrease HF symptoms [27]. This observation is consistent with suggested low flow rates for LA decompression for HFpEF patients by Abbasnezhad et al. [28]. Negative MLAP at rest with a pump support of 6 L/min suggests that the tested range might have been excessive, which aligns with findings from He et al. [29]. During simulated exercise, the HM3 reduced LAP but did not noticeably impact LVEDP. It provided a MLAP reduction of 44% at a pump support of 6 L/min. The LA unloading in rest and exercise is comparable to other rotary blood pump simulations with an LA inflow where LAP reductions of 35 – 70% at a maximum flow rate of 4 L/min are reported [17, 25].

While MLAP and MPAP decreased with increasing pump flow rates at rest, potentially relieving pulmonary congestion, the systolic LV pressure and the MAP increased with increasing pump flow rates in both rest and exercise. This may, in turn, cause further blood pressure increases and impact the VAD performance.

With increasing AOP, the aortic valve flow decreased, which indicates reduced aortic valve opening, causing increased LVESV and could lead to blood stagnation followed by thrombus formation in the LV. The HM3 in LA to Ao configuration has minimal impact on low flow rates, as the difference between LVESV and LVEDV decreases, but the LVESP increases as the left ventricular ejection fraction is preserved. The native cardiac output reduces and afterload increases with increasing pump flow rates, causing higher LVESV and therefore increased LVESP, leading to increased left ventricular loading and stroke work.

Based on the results of this study, a flow rate close to 1 L/min at rest and 6 L/min at exercise may be adequate for reducing LAP and LVEDP at rest while maintaining aortic valve flow. However, the HM3 is designed to operate at flow rates above 2.5 L/min and operating well-below the design point may increase the risk of thrombus formation within the pump. The addition of a physiological control algorithm to increase pump speed according to the activity of the patient seems desirable to achieve LA decompression in exercise. To reduce the risk of complications, a new device designed specifically for the HFpEF population might be needed.

The results of the virtual fitting study suggest that the size of the HM3 is inappropriate to fit in an LA to Ao configuration in 57% of the assessed HFpEF patients due to interference with other anatomical structures. It was shown that the heart geometries within the assessed patients vary substantially based on LA volume and minimal distance between the LA and descending Ao. The median LA volume in this study was higher than reported in previous studies (HFpEF: 178 mL vs 85 mL, HFrEF: 260 mL vs 104 mL) [19, 30, 31], which could be due to a difference in sample size, demographics and NYHA classification of the patients examined [30].

The increased LA volume in HFpEF favours the implantation of an MCS device in the LA, as the risk for inflow cannula obstruction and suction events decreases with increasing distance between the inflow cannula and the opposing LA wall [30, 31]. However, the LA typically has a smaller cavity and lower pressure compared to the LV, and there is a bigger pressure difference between the LA and the Ao compared to between the LV and the Ao. These differences might increase the risk of suction when the inflow cannula of an LVAD is implanted in the LA; thus, a gap spacer might be required to decrease the protrusion length [32]. The altered pressure differential between cannulated chambers may also render a pump in the LA to Ao configuration more susceptible to backflow, and thus, the pump design and potential pulsing algorithms should be carefully considered.

This study has several limitations. The in vitro cardiovascular simulator did not exactly replicate the mean hemodynamic values of the in vivo data, and oscillations in the recorded data may occur [33, 34] due to combination of noise in the sensors, compliance, inertia, and capabilities of the regulators. The presented model can only simulate upwards shifted LVEDPVR, and can therefore not represent every HFpEF phenotype. It did not model the baroreflex, which might impact the LVP and AOP. Moreover, this study was performed with constant SVR and PVR, constant chamber compliance and healthy RV contractility, which may not reflect actual clinical practice [35]. Therefore, these factors could have exaggerated the results in this study leading to values outside of the clinical range, especially the arterial and pulmonary pressures [36, 37] and ventricular volumes. Some of these limitations are addressed in a hybrid in vitro model of a HFpEF patient by He et al. [29]; however it is limited to a numerical simulation which does not capture the real-world fluid mechanics such as valvular dynamics [29]. While changes in the baseline condition may impact the results, this study is limited to two scenarios at 70 and 90 bpm. For a more thorough insight, more scenarios may be simulated with the presented model or other preclinical evaluation tools like numerical simulations and animal models, that may provide different results. The anatomical fitting study presented in this work was limited to 14 patients. Given the shown heterogeneity of the HFpEF population, bigger sample sizes should be investigated to allow a classification of suitable patient groups. Furthermore, surgical considerations should be made: the pump may be sewn into the LA, which has a lower wall thickness than the LV [30, 31]. Since the reconstruction of the heart geometries was based on CT images, which mainly distinguish between blood and tissue, the ventricular walls were not considered, but are negligible in this context due to their small thickness. For the development of the surgical implantation strategy, the wall thickness is a crucial factor and needs to be taken into consideration.

In conclusion, this study investigated the suitability of the HM3 for treating HFpEF patients. An in vitro assessment revealed that the HM3 in an LA to Ao configuration decreases the LAP and MPAP at the cost of increased LVP and AOP, therefore unloading the LA but increasing afterload in rest and exercise conditions. It was shown that the HM3 can fit into HFpEF patients without interfering with rigid anatomy but interfered with the aorta, pulmonary veins or right atrium in 57% of the assessed patients. This study suggests that a specialised pump designed for LA cannulation may be beneficial for unloading LA pressures of HFpEF patients and reducing their HF symptoms.

## Appendix 1

See Table 3.

**Table 3:** Patient data and virtual anatomical fitting results

Patient no.	Sex	Age [years]	Condition	LA volume [mL]	Distance LA-Ao [mm]	Impingement [mm]				Inflow cannula—LA wall [mm]	
					Minimal	Ao	PV	PA	RA	Minimal	Maximal
1	M	59	HFpEF	195	4.0	0.0	0.0	0.0	0.0	17.3	39.2
2	M	69	HFpEF	240	3.0	0.0	0.0	0.0	0.0	13.8	41.2
3	F	77	HFpEF	146	17.4	0.0	0.0	0.0	0.0	10.2	35.2
4	M	65	HFpEF	143	8.6	4.4	0.0	0.0	11.9	7.5	33.9
5	M	54	HFpEF	143	13.2	0.0	0.0	0.0	0.0	14.3	27.5
6	F	51	HFpEF	318	0.0	0.0	13.8	0.0	0.0	13.9	36.9
7	M	63	HFpEF	117	5.4	7.7	6.5	0.0	0.0	6.0	28.8
8	M	62	HFpEF	198	0.0	4.4	5.7	0.0	0.0	7.5	27.5
9	M	49	HFpEF	180	2.2	0.0	0.0	0.0	0.0	16.6	47.4
10	M	59	HFpEF	184	5.1	0.0	3.8	0.0	0.0	15.0	31.2
11	F	72	HFpEF	175	3.6	6.6	11.9	0.0	0.0	13.4	38.1
12	F	69	HFpEF	122	3.3	4.7	9.8	0.0	0.0	12.2	27.8
13	F	74	HFpEF	240	4.4	0.0	0.0	0.0	0.0	13.5	50.5
14	F	76	HFpEF	161	1.7	0.0	12.3	0.0	0.0	16.9	42.7
*Median*			HFpEF	178	3.8	0.0	1.9	0.0	0.0	13.6	36.1
15	M	67	HFrEF	152	10.9	–				–	
16	F	44	HFrEF	131	17.8	–				–	
17	M	66	HFrEF	325	0.0	–				–	
18	M	65	HFrEF	264	3.1	–				–	
19	M	50	HFrEF	267	1.6	–				–	
20	M	30	HFrEF	287	0.0	–				–	
Median			HFrEF	266	2.3	–				–	

*LA* left atrium, *Ao* aorta, *PV* pulmonary veins, *PA* pulmonary artery, *RA* right atrium
